# The Kentucky State Medical Society

**Published:** 1891-07

**Authors:** 


The Kentucky State Medical Society held one of the most successful meetings in its history at Lexington, May 27 and 28, 1891. Dr. McMurtry, of Louisville, made an interesting and able report on Abdominal Surgery, and Dr. Arch. Dixon, of Henderson, read a paper of great interest on The Use and Abuse of the Surgical Probe. The Society honored itself in electing Dr. H. Brown, of Hustonville, as president for the ensuing year. Dr. David Barrow, of Lexington, as chairman of the committee of arrangements, contributed largely to the success of the meeting by his excellent management of all details pertaining to his office, while the banquet was especially conspicuous for good form, elegant service, and all that goes to make such an occasion enjoyable.

